# The unhappy postdoc: a survey based study

**DOI:** 10.12688/f1000research.12538.2

**Published:** 2018-05-02

**Authors:** Amir Grinstein, Roi Treister

**Affiliations:** 1 Northeastern University, 360 Huntington Avenue, Boston, MA 02115, USA; 2 VU University Amsterdam, De Boelelaan 1105, Amsterdam, 1081 HV, Netherlands; 3 Haifa University, 199 Abba Khoushy Avenue, Haifa, 3498838, Israel; 4 Massachusetts General Hospital & Harvard Medical School, 55 Fruit St, Boston, MA 02114, USA

**Keywords:** Postdoc, post-doctorate, well-being, academic career

## Abstract

**Background:** The emerging public discourse about the “broken” postdoc system is mostly conceptual (with several recent exceptions). The current work offers an attempt to quantify postdocs’ perceptions, goals, and well-being.

**Methods: **A survey of 190 postdocs in North America.

**Results: **This article first reveals a surprisingly unhappy postdoc community with low life satisfaction. Second, it demonstrates how over the course of the fellowship many postdocs lose interest in the goal of pursuing a tenure track academic position (~20%) or in recommending the postdoc track to others (~30%). Finally, we find that among a large number of factors that can enhance life satisfaction for postdocs (e.g., publication productivity, resources available to them) only one factor stood out as significant: the degree to which atmosphere in the lab is pleasant and collegial.

**Conclusions: **Our findings can stimulate policy, managerial, and career development improvements in the context of the postdoc system.

## Introduction

Post-doctorate fellows (i.e. postdocs) are a major force in advancing scientific research, and often are the driving force behind successful labs, especially in the bio-medical area. Not only the sheer number of postdocs is on the rise – the number of postdocs has tripled since 1979 ( Gould, 2015) – but also their research projects require heavier funding as the costs of conducting top-quality research have increased ( Davis, 2009; Stephan, 2012; Xuhong, 2013). A common assumption is that PhDs pursue a postdoc position in an academic research institution to enhance their research skills and reputation, which in turn increases their chances of obtaining the ultimate goal: a tenure track academic appointment. While this is a worthy goal to pursue, and there is no doubt that a postdoc position is often key for a future academic appointment, there are growing concerns that the postdoc system is broken and unsustainable ( Alberts *et al.,* 2014; Gould, 2015). Such concerns are mostly heard from postdocs ( Powell, 2015; Smaglik, 2016). Conversely, the academic establishment benefits from maintaining the status quo because of the value in skilled employees like postdocs – that are relatively non-costly and require minimal training and supervision ( Smaglik, 2016). However, the benefits from a postdoc career for the postdocs themselves are becoming much less evident. Under unstable economic conditions tenure track academic appointments become tremendously difficult to obtain. Recent evidence from the UK, for example, suggests that of 100 science PhD graduates, about 30 will go on to postdoc research, but just 4 will secure permanent academic posts with a significant research component ( Nature editorial, 2014). In the US, the situation is slightly better: 65% of all PhD holders follow the postdoc path, with this estimation increasing to 80% among bio-medical PhDs but only 15–20% of those by some sources, and 8% by others, gain a tenure track position ( Gould, 2015; Kahn & Ginther, 2017; Sauermann & Roach, 2016). Moreover, postdocs that are not able to achieve an academic appointment often become over-qualified for industry positions while losing alternative higher compensation (salaries in the industry) and often putting their personal life (marriage, children) on hold ( Kahn & Ginther, 2017).

It takes between 12–18 years of academic training to get into the entry level of an academic tenure track position ( Gould, 2015). This very long training process has economic, social, and individual well-being costs. These heavy costs are often complemented with the significant uncertainty surrounding the likelihood of obtaining a tenure track academic appointment. Overall therefore, it might be very useful for potential postdocs to develop a more critical view of the traditional academic postdoc track and of their career choices after completing their PhD.

While discussions of the postdoc reality have been attracting growing attention (e.g., Alberts *et al.,* 2014; Gould, 2015; Miller & Feldman, 2015; Powell, 2015; Smaglik, 2016), they have mostly focused on the policy level and lacked empirical assessments at the individual postdoc level (with a few exceptions mentioned next). Our aim is to empirically study the perspective of postdocs, to better understand their goals and perceptions, and to be able to promote evidence-based career choices by PhDs considering the postdoc path. We follow recent empirical efforts conducted by Gibbs *et al.* (2015); Miller & Feldman (2015); Sauermann & Roach (2016) and Faupel-Badger *et al*. (2017). Specifically, we pose, at the individual level, the following unanswered research questions:

Given the complex reality postdocs face today

(1) How satisfied are current postdocs?
(2) How likely are they to change – over the course of their fellowship – their key career goal of (typically) obtaining a tenure track appointment?

## Methods

To answer our research questions, we conducted a survey of postdocs in North America during the second half of 2015, mostly in the bio-medical and physical sciences. We emailed the survey to 29 leading postdoc associations in North America (e.g., National Postdocs Association, Rockefeller Postdocs Association, Johns Hopkins Postdoctoral Association) asking them to distribute it among their members. Overall 190 postdocs completed the survey ^1^. Respondents’ anonymity was kept. The majority of respondents were positioned in the U.S, with 6 participants from Europe, Asia and Africa ^2^. Table 1 summarizes key characteristics of the surveyed postdocs. The survey, data, and list of postdoc associations targeted can be found as a supplementary files ( Dataset 1, Supplementary File 1, Supplementary File 2).

**Table 1.** Key characteristics of the surveyed postdocs. Different n are due to missing values (where participants did not provide information). Percentages are of valid cases.

	# of Postdocs fellowships	Duration of postdoc fellowship(s) (years)	# of total publications	# of publications during the postdoc fellowship(s)	Discipline	Age	Gender
	One 70.0%	≤1 21.3%	0 2.2%	0 33.1%	Biology 43.6%	<30 9.5%	Female 55.1%
	Two 26.3%	2–4 50.0%	1–3 12.4%	1 19.7%	Neuroscience 21.8%	30–39 78.3%	Male 44.9%
	Three 3.2%	5–8 24.1%	4–10 50.6%	2 10.7%	Medicine 10.9%	40–49 11.5%	
	Four 0.5%	>8 4.7%	>10 34.8%	3 14.0%	Engineering 4.2%	>50 0.7%	
				>4 22.5%	Chemistry 3.0%		
					Environmental science 1.2%		
					Physics 0.6%		
					Mathematics 0.6%		
					Statistics 0.6%		
					Other 13.5%		
n	190	178	178	178	165	148	147
Min-Max	1–4	0.5–18	0–65	0–20	-	27–52	-
Mean	1.34	3.38	9.26	2.28	-	34.89	-

Survey response A data set including the response of 190 North American postdocs. The .zip file contains dataset in .sav and .xls formats. Click here for additional data file. Copyright: © 2018 Grinstein A and Treister R 2018 Data associated with the article are available under the terms of the Creative Commons Zero "No rights reserved" data waiver (CC0 1.0 Public domain dedication).

In the survey we were especially interested in collecting data regarding satisfaction levels of postdocs as well as their career goals dynamics while accounting for variety of factors that may impact these outcomes such as number of publications and atmosphere in the lab.

## Results

Life satisfaction was quantified by five items, each reported on a 1–7 scale, based on the established Diener *et al.*’s (1985) Satisfaction with Life scale (that showed very high reliability as measured by Cronbach’s α=.90): “In most ways my life is close to my ideal,” “The conditions of my life are excellent,” “I am satisfied with my life,” “So far I have gotten the important things I want in life,” “If I could live my life over, I would change almost nothing”. A first set of findings suggests that postdocs are far from being satisfied with their current situation in life with a mean of 4.47 (SD=1.46). Furthermore, 30% of participants demonstrate lower than the mid-point (=4) satisfaction levels. Considering that prior research using the same satisfaction scale typically suggests a positive bias of people when asked about life satisfaction ( Abdallah *et al.*, 2009; Diener *et al.*, 1985), our results demonstrate a surprisingly low well-being among people that are one step away from their “dream” appointment position. Indeed, prior work on life satisfaction of other types of skilled trainees that are roughly at the same age group (e.g., medical students) report much higher satisfaction levels (not lower than 5.2 (e.g., Kjeldstadli *et al.*, 2006; Samaranayake & Fernando, 2011). While locating our finding of a 4.47 mean on the Satisfaction with Life scale suggests this score falls under the “slightly satisfied” category ( Pavot & Diener, 1993) the finding is the lowest compared to all other student groups in developed countries or professionals (apart from “elderly caregivers”) as reported in Table 1 in Pavot & Diener (1993). In fact, the postdocs fall significantly behind the general US population, where many postdocs reside ( Abdallah *et al.*, 2009). According to the Pavot & Diener (1993), life satisfaction in the developed world is likely to be “slightly satisfied” as a starting point. With this context in mind, our results actually show a surprisingly low satisfaction with life scores of postdocs.

Interestingly, out of a list of potential explanatory variables of life satisfaction among postdocs – inspired by prior work (e.g., Faupel-Badger *et al*., 2017; Miller & Feldman, 2015) – including postdocs’ demographic characteristics (age, gender), personal characteristics (number of postdocs, total years in postdoctoral positions, discipline), publication productivity (number of publications, number of publications based on the postdoc), PI characteristics (number of publications, frequent interaction of the postdocs with the PI), and lab characteristics (value of equipment, number of postdocs) – only one factor showed significant relationship with life satisfaction: atmosphere in the lab (r=.247, p=.002). This measure included five items on a 1–7 scale, inspired by Moos’ (2008) Work Environment Scale’s Peer Cohesion sub-dimension aiming to understand how friendly and supportive employees are to each other (α=.80 “The atmosphere in the lab is/was very pleasant,” “I am/was happy to go to work in the morning,” “I view/viewed my lab colleagues as friends,” “I often have/had social interactions with my lab colleagues outside the lab,” “I often collaborate/collaborated on joint projects with my lab colleagues”). Table 2 reports the correlation matrix between the key variables.

**Table 2.** Correlation matrix.

	Total years in postdoctoral positions	Gender	Age	Life satisfaction	Atmosphere in the lab	Discipline	# of total publications	# of publications during the postdoc fellowship(s)	# of publications of PI	Frequent interaction with PI	Estimated value of equipment in lab	# of postdocs in lab
**Total years in** **postdoctoral** **positions**	1											
**Gender**	.116	1										
**Age**	.621 **	.197 *	1									
**Life** **satisfaction**	-.031	-.046	-.062	1								
**Atmosphere** **in the lab**	-.014	.037	.079	.247 **	1							
**Discipline**	-.112	.170 *	-.007	.018	.084	1						
**# of total** **publications**	.478 **	.076	.123	.000	-.043	.185 *	1					
**# of** **publications** **during the** **postdoc** **fellowship(s)**	.483 **	.113	.208 *	.039	.044	.093	.595 **	1				
**# of** **publications** **of PI**	.044	.121	-.063	.046	-.003	.025	.106	.139	1			
**Frequent** **interaction** **with PI**	.028	-.049	-.035	-.106	-.208 **	-.034	.003	-.056	.229 **	1		
**Estimated** **value of** **equipment** **in lab**	.114	.169 *	.102	.140	.316 **	.008	.042	.117	.214 **	-.077	1	
**# of** **postdocs in** **lab**	.061	.069	-.077	.012	.055	-.213 **	-.080	-.054	.543 **	.272 **	.317 **	1

Gender coding = 1-female, 2-male; Discipline coding= 0-bio-medical, 1-other. *Correlation is significant at the .05 level; **Correlation is significant at the .01 level.

We support this analysis with a more rigorous regression analysis. Given we did not have a-priori predictions we followed an exploratory approach and tested multiple regression analyses including various combinations of the factors that may drive postdocs’ life satisfaction. The best performing model (R ^2^ = 8.5%, F = 2.11, p = .056), reported in Table 3, demonstrated that atmosphere in the lab was the only significant factor positively affecting postdocs’ life satisfaction (β = .250, p = .005).

**Table 3.** Drivers of postdocs’ life satisfaction – regression analysis.

	*Life satisfaction*	
	Standardized estimates	*p*-value
Atmosphere in the lab	.250	.005
Gender	-.053	.550
Age	-.064	.464
Discipline	-.008	.930
# of total publications	.011	.900

Gender coding = 1-female, 2-male; Discipline coding= 0-bio-medical, 1-other.

A key consequence of such lack of satisfaction is represented in participants’ responses to a question wondering whether the postdocs will likely to recommend the postdoc track to others who are considering it. Only 28.4% “agreed” or “definitely agreed” to recommend the postdoc path to others.

**Figure 1.**
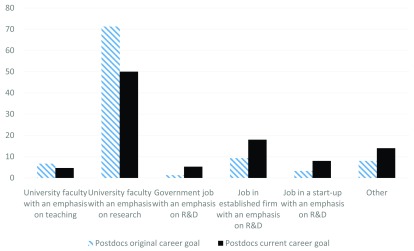
Postdocs career goal change over the span of their fellowship.

Another finding is that many postdocs are re-considering or re-considered their career goals during their fellowship. We asked participants to share with us their main career goal when starting their postdoc and also at the point of the survey. Options included (a) university faculty with an emphasis on teaching, (b) university faculty with an emphasis on research, (c) government job with an emphasis on R&D, (d) job in an established firm with an emphasis on R&D, (e) job in a start-up with an emphasis on R&D or (f) other. The results (summarized in Figure 1) shows a shift from a goal focusing on academic tenure track position to other goals – mostly industry positions. In our sample, 71.3% of the postdocs began their fellowship with the goal of pursuing a tenure track academic position. When the survey was conducted only 50% maintained a similar career goal. The key career change involves a focus on the industry (established R&D company or a start up) – from 9% and 3%, to 18% and 8%, respectively. These findings are overall consistent with other research into career trends of postdocs (e.g., Mason *at al*., 2016; Roach & Sauermann, 2017; Sauermann & Roach, 2016).

## Discussion

Many postdocs are facing a well-being paradox. On the one hand, a postdoc position is a very meaningful step towards achieving many postdocs’ central goal of obtaining a tenure track appointment. On the other hand, however, the growing realization that these positions are more scarce than ever before, as well as the long, frustrating, and not always rewarding postdoc journey significantly damages the well-being and satisfaction of many postdocs.

Our empirical analysis is a valuable first step in documenting and reflecting on the notion of the well-being paradox of postdocs. Unhappy postdocs is not a good recipe for sustainable success of the postdoc system and for advancing top-tier scientific work. This means – from the perspective of policy makers, university administration, and lab leaders – that there is value in better understanding and catering to the well-being and needs of individual postdocs. Our finding that a key aspect of postdocs’ life satisfaction involves a positive atmosphere in the lab attests to the importance of “soft” factors in creating a sustainable and successful postdoc system, not necessarily science or monetary related factors. This finding corresponds well with prior research that shows how postdocs’ experience can be improved through interpersonal, lab-level interventions and mentorship rather than more tangible issues of pay and benefits ( Davis, 2009; Miller & Feldman, 2015; Scaffidi & Berman, 2011). Such insight can help lab leaders in postdoc recruitment efforts and in optimizing the postdoc experience.

Further, postdocs and prospective postdocs (mostly PhDs) should consider adopting a more critical view of the traditional academic postdoc track. This, sometimes referred to in the literature as “better planning” ( Sauermann & Roach, 2016) may lead to seriously considering all available career options including an industry position following the PhD, an industry postdoc, or a combined academic-industry postdoc. A recent finding that even after achieving a tenure-track academic position, many assistant professors are unhappy ( Nature editorial, 2016), could be another catalyst for postdocs to re-think their career goals. Overall, these trends may require the industry to be more proactive in establishing postdoc positions while requiring the academic system to be more open and flexible with respect to non-academic postdocs and collaboration with the industry. A noteworthy trend however is that some industry actors are actually hiring fewer people for research positions and that the bio-medical workforce is a shrinking component. Reasons include the increasing practice of R&D outsourcing ( Heggeness *et al*., 2016; Mason *et al*., 2016).

Our work suffers from some limitations that may guide future work. First, we collected data through postdoc associations. These associations may not necessarily include all postdocs at an institution and may be biased towards the unionized members. Future work may aim also at independent postdocs while controlling for postdoc association membership. Second, the individual postdocs surveyed have different cultural backgrounds, which may impact, for example, satisfaction tendencies and survey response styles. Future work can examine the moderating role of culture on the effects we find. Third, sample selection may be an issue, leading the most dissatisfied postdocs to participate in the survey. Another form of sample selection may be the result of our sample being oriented towards bio-medical postdocs that have relatively low chances of getting a tenure track position ( Ghaffarzadegan *et al*., 2015; Larson *et al*., 2014). This may lead their life satisfaction to be on average lower relatively to other postdocs. Future work should increase and diversify the sample to further minimize selection concerns. Finally, our explanations to postdocs’ life satisfaction captures multiple dimensions including personal, lab and the PI’s characteristics. Still the low life satisfaction may be attributed to alternative factors such as the misalignment between postdocs’ changing preferences for specific job attributes on the one hand, and the nature of the academic research career itself on the other ( Roach & Sauermann, 2017). Future work may further examine this and additional factors.

Overall, our work helps to better understand the well-being of postdocs and its drivers. It provides empirical support to the idea that the current postdoc system is broken and that postdocs are paying a price in well-being terms. Any successful change in the postdoc system would need to enhance postdocs’ well-being and it is our hope that our findings stimulate policy, managerial, and career development improvements that can be pursued.

## Data availability

The data referenced by this article are under copyright with the following copyright statement: Copyright: © 2018 Grinstein A and Treister R

Data associated with the article are available under the terms of the Creative Commons Zero "No rights reserved" data waiver (CC0 1.0 Public domain dedication). 

**Dataset 1. Survey Response.** A dataset including the response of 190 North American postdocs. The .zip file contains dataset in .sav and .xls formats. 10.5256/f1000research.12538.d202390 ( Grinstein & Treister, 2018)

## Ethics and consent

The first author’s university ethics committee (Northeastern University’s Institutional Review Board) has approved the project “The Unhappy Postdoc” (IRB#: 15-05-01). Written informed consent was obtained from all participants.

## Notes

^1^Unfortunately we did not get access to the associations’ member lists or to information about them so we lack data on the overall number of survey invitations sent. The overall sample frame according to the latest available NSF Survey of Graduate Students and Postdoctorates in Science, Engineering and Health is 64,000 in 2015 ( https://www.nsf.gov/statistics/2017/nsf17309/nsf17309.pdf).

^2^These 6 postdocs were part of the associations and have recently moved back to their home country.

## Supplementary Material

**Supplementary file 1:** A survey that was distributed among 190 North American postdocs.

**Supplementary file 2:** List of postdoc associations targeted legend: a list of 29 central postdoc associations in North America that helped distribute the survey among their members.
