# Supplementary material for: The unhappy postdoc: a survey based study
Source: F1000Res. 2018 May 2;6:1642. Originally published 2017 Sep 5. [Version 2] doi: 10.12688/f1000research.12538.2 (PMC5958315; doi:10.12688/f1000research.12538.2)
Supplement: Supplementary file 2 [file f1000research-6-16131-s0001.tgz › bb9ba5c1-a933-4e15-9ffa-971180c34754.pdf]

Default Question Block

<u>Research on Post-Doctorate (Post-Doc) Goals and Conditions for Success</u>
<u>Research Participant Information and Consent Form</u>

You are asked to participate in a research study conducted by Professor Amir Grinstein, from the Department of Marketing at Northeastern University. Researchers are required to provide a consent form to inform you about the study, to convey that participation is voluntary, to explain risks and benefits of participation, and to empower you to make an informed decision. You should read the information below and feel free to ask the researchers any questions you may have.

1. PURPOSE OF RESEARCH
You are being asked to participate in a research study on post-docs goals and conditions for success. You have been selected as a possible participant in this study because you are a post-doc or was a post-doc in recent years, and over 18 years of age. From this study, the researchers hope to learn about the goals and beliefs of post-docs and about the conditions which are optimal for successful post-doc fellowships. The research will be useful for post-doc fellows, PhD students, and policy makers in academia.

2. WHAT YOU WILL DO
Your participation in this study will take about 10-12 minutes. You will be asked to complete a survey about your post-doc fellowship.

3. POTENTIAL BENEFITS
There are no direct benefits to you for participating in the study. Participants that are interested in getting a copy of the study results should please contact Professor Amir Grinstein at a.grinstein@neu.edu. Your participation in this study may contribute to the understanding of the conditions for a successful post-doc fellowship.

4. POTENTIAL RISKS
The possible risks or discomforts of the study are minimal if any. If you feel uncomfortable, at any time you can withdraw from the study.

5. PRIVACY AND CONFIDENTIALITY
The data for this project are being collected anonymously. The data are being coded by assigning a random number for each participant. Therefore neither the researchers nor anyone else will be able to link data to you and your privacy will be maintained throughout the survey and data analysis. The data for this project and information about you will be kept confidential to the maximum extent allowable by law. The data will be stored on an external hard drive in the offices of the Principal Investigator at Northeastern campus, behind a locked door. Only the Principal Investigators will have access to the data. The results of this study may be published or presented at professional meetings, but the identities of all research participants will remain anonymous.

6. YOUR RIGHTS TO PARTICIPATE, SAY NO, OR WITHDRAW
Participation in this research project is completely voluntary. You have the right to refuse to participate in the study. You may change your mind at any time and withdraw from the study; you are also allowed to skip a question if you are uncomfortable answering it.

7. COSTS AND COMPENSATION FOR BEING IN THE STUDY
There is no compensation involved in this study.

8. CONTACT INFORMATION FOR QUESTIONS AND CONCERNS
If you have concerns or questions about this study, such as scientific issues, how to do any part of it, or to report any issue, please contact Professor Amir Grinstein at a.grinstein@neu.edu.

If you have any questions regarding electronic privacy, please feel free to contact Mark Nardone NU's Director of Information Security via email at privacy@neu.edu

If you have any questions about your rights in this research, you may contact Northeastern University's Office of Human Subject Research Protection, 490 Renaissance Park, Northeastern University, Boston, MA 02115. Tel: 617.373.4588, Email: irb@neu.edu. You may call anonymously if you wish.

This study has been reviewed and approved by the Northeastern University Institutional Review Board (#15-05-01).

9. DOCUMENTATION OF INFORMED CONSENT
Clicking the "I agree" button below indicates your voluntary agreement to participate in this research project.

- ○ I agree to participate in this research

Professional experience

The following questions refer to your professional experience as a post-doc.

If you are no longer a post-doc, please refer to your time as a post-doc.

I am taking/took part in

- A single post-doc fellowship
- Two post-doc fellowships
- Three post-doc fellowships
- Four post-doc fellowships or more

I am taking/took part in

- An academic post-doc fellowship
- A combined academic and industry post-doc fellowship
- Started an academic post-doc fellowship and then moved to an industry post-doc fellowship (or vice versa)
- An industry post-doc fellowship

How many years since you began your (first) post-doc fellowship?

How many peer-reviewed publications do you have?

If you have publications, on how many of these you are the first author?

How many peer-reviewed publications you were able to publish based on your post-doc fellowship(s) [during or following your post-doc]?

Please include ONLY those publications originated from your post-doc projects.

If you have publications based on your post-doc, on how many of these you are the first author?

Please report the names of the top 3 journals you published in based on your post-doc fellowship(s):

Name of Journal

Name of Journal

Name of Journal

Do you have patents that have been filed, listing you as an inventor during/based on your post-doc fellowship(s)?

If you have patents, please report how many

In what discipline is/was your post-doc fellowship?

If "other", please elaborate:

In what department is/was your post-doc fellowship?

Organizational atmosphere

The following questions refer to your post-doc lab (please focus on your RECENT post-doc fellowship)

Please share your agreement level with the following statements

	Definitely do not agree	Do not agree	Somewhat do not agree	Neither do not agree nor agree	Somewhat agree	Agree	Definitely agree
The atmosphere in the lab is/was very pleasant	○	○	○	○	○	○	○
I am/was happy to go to work in the morning	○	○	○	○	○	○	○
I view/viewed my lab colleagues as friends	○	○	○	○	○	○	○
I often have/had social interactions with my lab colleagues outside the lab	○	○	○	○	○	○	○
I often collaborate/collaborated on joint projects with my lab colleagues	○	○	○	○	○	○	○
I would recommend my colleagues/friends to apply for a post-doc position in my lab	○	○	○	○	○	○	○

Lab resources

The following questions refer to your post-doc lab resources

What is your main PI age? (you can make your best estimation)

[▼]

How many peer-reviewed publications does your PI have?

[▼]

What is/was the frequency of your one-on-one personal meetings with your PI (please account only the meeting that exceed 20 minutes)

[▼]

How many full time post-doc fellows are currently working in your laboratory/worked in your laboratory during your post-doc?

[▼]

How diverse is/was the group of post-docs that populate/d your lab?

	Not diverse at all	Not diverse	Somewhat not diverse	Neither not diverse nor diverse	Somewhat diverse	Diverse	Highly diverse
In terms of country of origin (where people were born and spent the majority of their early life)	○	○	○	○	○	○	○
(where people were born and spent the majority of early life)	○	○	○	○	○	○	○

To the best of your knowledge and relatively to other post-doc labs in your research area, your laboratory equipment value (mostly consider big devices such as microscopes etc, don't count pipettes) is

	Very low	Low	Somewhat low	neither low nor high	Somewhat high	High	Very high
Lab equipment value is	○	○	○	○	○	○	○

Question regarding your goals and beliefs

The following questions address your goals and beliefs

When you <u>began</u> your post-doc position, which of the following career goals did you aspire to?

If "other", please elaborate:

Currently, which of the following career goals do you aspire to?

If "other", please elaborate:

Please share your agreement level with the following statement

	Definitely do not agree	Do not agree	Somewhat do not agree	Neither do not agree nor agree	Somewhat agree	Agree	Definitely agree
I would recommend my friend or colleague to pursue a post-doc position	○	○	○	○	○	○	○

In your search for academic position do/did you limit yourself to positions at institutes in your country of origin *(where you were born and spent the majoirty of your early life)*?

In your search for an academic position, what are/was your geographical priority?

In the case you are pursuing an academic, tenure-track position, how do you perceive your chances of getting such a position?

If your initial intentions to pursue a professorship position changed, what are the main reasons for the change?

If "other", please elaborate:

What is your current position?

If "other", please elaborate:

Please share your agreement level with the following statements regarding current satisfaction with life

	Definitely do not agree	Do not agree	Somewhat do not agree	Neither do not agree nor agree	Somewhat agree	Agree	Definitely agree
In most ways my life is close to my ideal							
The conditions of my life are excellent							
I am satisfied with my life							
So far I have gotten the important things I want in life							
If I could live my life over, I would change almost nothing							

Demographics

The following questions address general and demographic issues

Please indicate your gender

Please indicate your age

What was your marital status when starting the FIRST post-doc?

If "other", please elaborate:

What is your country of origin? (*where you were born and spent the majority of your early life*)

In what country did you receive your PhD?

In what country is/was your post-doc?

In what country is your current position (in the academic world or industry)?

What is your highest academic degree?

If "other", please elaborate:

Thank you for your participation!

If you want a copy of the study results please email Professor Amir Grinstein at a.grinstein@neu.edu
