# Supplementary material for: The unhappy postdoc: a survey based study
Source: F1000Res. 2018 May 2;6:1642. Originally published 2017 Sep 5. [Version 2] doi: 10.12688/f1000research.12538.2 (PMC5958315; doi:10.12688/f1000research.12538.2)
Supplement: Supplementary file 3 [file f1000research-6-16131-s0002.tgz › d985b5ea-387a-49fe-902a-6cf43e10b296.pdf]

Supplementary file 2: List of postdoc associations targeted legend: a list of 29 central postdoc associations in North America that helped distribute the survey among their members.

#	**Postdoc Association**
1	University of California, San Diego
2	Johns Hopkins
3	Rockefeller University
4	Albert Einstein College
5	Princeton
6	University of California, Berkeley
7	North Carolina State University
8	University of Washington
9	University of Alabama
10	Purdue University
11	Los Alamos
12	Karolinska Institute
13	University of Calgary
14	University of Pennsylvania
15	National Postdoctoral Association
16	Caltech
17	University of Chicago
18	University of North Carolina
19	University of Pittsburgh
20	Dartmouth
21	University of Georgia
22	University of California, Davis
23	Cornell
24	Yale
25	Canadian Post Doc Association
26	MGH Harvard
27	Max Plank Society
28	Harvard Medical School
29	Science Abroad
