# Supplementary material for: The unhappy postdoc: a survey based study
Source: F1000Res. 2018 May 2;6:1642. Originally published 2017 Sep 5. [Version 2] doi: 10.12688/f1000research.12538.2 (PMC5958315; doi:10.12688/f1000research.12538.2)
Supplement: Peer review history

# Peer review: Referee response for version 2

**DOI:** 10.5256/f1000research.16131.r33660

**Published:** 2018-08-13

**Authors:** Gary S. McDowell

**Affiliations:** 1 The Future of Research Inc., 848 Brockton Avenue, Abington, MA, USA

The literature review is somewhat improved covering the background of studies such as this. A larger study that has taken place, which still required the self-selection of postdocs to complete the survey, has been discussed elsewhere ^1^ and it could be worth comparing this data with that.

 I still however have concerns about the claims this data tries to make, particularly on such a small and biased sample, sharing the concerns of Nick Riddiford's review. This dataset may be a useful preliminary indicator of some of the factors important to postdoc satisfaction, and while I am reassured that much of what appears supports conclusions elsewhere, I am very concerned at trying to draw meaningful conclusions from the work.

 In addition in the Supplementary data the list of postdoc associations in North America includes groups such as "Science Abroad", an organization of Israeli scientists abroad (which may address the comment in Nick Riddiford's report about the large proportion of Israeli-identifying participants in the survey data), which may not be limited to North America, and the Karolinska Institute, which is in Sweden, in Europe.

I have read this submission. I believe that I have an appropriate level of expertise to confirm that it is of an acceptable scientific standard, however I have significant reservations, as outlined above.

## References

[1] McConnellSWestermanEPierreJHecklerESchwartzN: Career Choice, Gender, and Mentor Impact: Results of the U.S. National Postdoc Survey. *bioRxiv*.2018; 10.1101/355511 10.1101/355511 PMC629878330561332

# Peer review: Referee response for version 2

**DOI:** 10.5256/f1000research.16131.r33663

**Published:** 2018-07-05

**Authors:** Nick Riddiford

**Affiliations:** 1 Institut Curie, Paris, France

While a number of changes have been made to address the concerns I and others raised, I find the revised version too weak, and biased, to recommend for indexing.

 Most importantly, on inspecting **Dataset 1 - Survey Response**, I see that 88% of respondents who answered Q41 ( **country of origin**), and 94% of respondents who answered Q43 ( **country of PhD**) gave the response **Israel**. This to me suggests that there is a huge distribution bias that needs to be seriously confronted.

 To attempt to make broad claims about postdocs, based on such a biased distribution and in conjunction with otherwise fairly uninspiring data, is flawed, and misleading. 

 In addition to this bias, the correlation is still poorly explored, and the methods are not explained in detail at all, and are instead only briefly mentioned in the results section. 

 Considering these points, I currently cannot recommend this article for indexing.

I have read this submission. I believe that I have an appropriate level of expertise to state that I do not consider it to be of an acceptable scientific standard, for reasons outlined above.

# Peer review: Referee response for version 2

**DOI:** 10.5256/f1000research.16131.r33662

**Published:** 2018-06-11

**Authors:** Jennifer M. Miller

**Affiliations:** 1 USC Sol Price School of Public Policy, University of Southern California, Los Angeles, CA, USA

I appreciate the revisions that have been made to address concerns in my earlier review and now recommend the article for indexing.

I have read this submission. I believe that I have an appropriate level of expertise to confirm that it is of an acceptable scientific standard.

# Peer review: Referee response for version 2

**DOI:** 10.5256/f1000research.16131.r33661

**Published:** 2018-05-17

**Authors:** Navid Ghaffarzadegan, Ran Xu

**Affiliations:** 1 Grado Department of Industrial & Systems Engineering (ISE), Virginia Tech, Blacksburg, VA, USA; 2 Virginia Tech, Blacksburg, VA, USA

We thank the authors for carefully revising the paper. The revisions are sufficient. The paper brings new insights into the problem of job dissatisfaction and unhappiness among postdoctoral fellows, and it can have major policy implications.

We have read this submission. We believe that we have an appropriate level of expertise to confirm that it is of an acceptable scientific standard.

# Peer review: Referee response for version 1

**DOI:** 10.5256/f1000research.13576.r26675

**Published:** 2017-10-16

**Authors:** Jennifer M. Miller

**Affiliations:** 1 USC Sol Price School of Public Policy, University of Southern California, Los Angeles, CA, USA

This review will focus a critical eye on three issues, integration with existing literature, survey methodology, and use of the Satisfaction with Life Scale (SWLS). The review then acknowledges some points of agreement and support for the authors and ultimately recommends significant revision for publication, with some reservations.

 The abstract begins with the implication that the public discourse about postdocs is not informed by empirical work. The implication that empirical work is missing is a red flag to me *whenever* I encounter it, not only when it is my own empirical work that is being overlooked. Yet I do believe that our paper (Miller & Feldman, 2015) that uses the Science and Engineering PhD and Postdoc Survey (SEPPS) conducted by Sauermann and Roach to study dissatisfaction with postdoc appointments could have informed the design and interpretation of this study. So at the risk of being a caricature of reviewers who plug their own work, I will begin by pointing the authors toward this paper, which also contains a summary of prior research on postdoc satisfaction. The Scaffidi and Berman (2011) paper is also very relevant to the topic.

 I was pleased to see that the authors had cited Davis’s 2009 chapter analyzing the Sigma Xi survey of postdocs, as this has been some of the most influential empirical work on the postdoc experience. As a methodological step to avoid this type of disconnect from prior literature, I would recommend the practice of using Google Scholar’s “cited by” feature to look at the studies that have cited this chapter. It will connect the authors to even more recent and relevant empirical work.

 Further review of existing literature would also have been helpful to the authors in their use of the SWLS. Diener and colleagues have published a number of subsequent review articles (including but not limited to Diener & Pavot 1993 and Pavot & Diener 2008), but this study relies on the original 1985 paper.

 The review will now turn to issues of interpretation related to the survey data and measurement scales.

 Interpretation of the survey data was complicated by the absence of a correlation table. It was not entirely clear, but it appears that conclusions are drawn from correlations, rather than regression analyses that more carefully model the relationship to satisfaction by including control variables. I recommend the authors clearly explain the analyses performed and provide a correlation table.

 I have two concerns regarding the representativeness of the sample. First, the authors say the survey was distributed by postdoc associations *to their members*. Postdoc associations do not necessarily include all postdocs at an institution and often serve as agents of change, sometimes including formal unionization efforts. This is a limitation that would discourage me from drawing policy conclusions from the study. Postdoc association membership lists may overrepresent dissatisfied postdocs. Of course, it is also possible that at least some postdoc associations have a broader mailing list. It is even possible that not all postdoc associations distributed the survey at all. With only 29 associations, it seems likely it would be feasible to contact them to confirm distribution and to give some consideration to their mission statements.

 Second, in analyzing survey data, it is a questionable choice to include only complete cases. Various techniques for imputation allow the researchers to avoid discarding the valuable data from incomplete cases. The fact that age and gender were missing from over 20% of cases, while other variables were provided more completely, suggests that there may be some bias introduced by discarding missing data. Response bias also tends to overrepresent those with strong feelings and opinions.

 The likelihood for sample bias due to the reliance on postdoc associations is my main concern with the study as a whole. This cannot be remedied but can be acknowledged as an important limitation.

 Comparison of postdocs’ SWLS scores with early studies of relevant populations was complicated by the fact that the authors report the mean (4.47 on a 1-7 scale), while apparently other studies tend to report the total scale score (5-35). On page 2, the authors refer to 4 as the median, when 4 is more accurately described as the midpoint of the scale. Diener & Pavot (1993) are careful to interpret findings relevant to their scale anchors. A mean of 4.47 indicates a neutral to slightly satisfied group.

 But taking into account confidence intervals, it is not clear to me that the satisfaction of postdocs is significantly worse than that found by Diener & Pavot in 1993 for several groups of US college students. Given the cultural variation in reporting life satisfaction, the absence of nationality data also complicates interpretation of the scale (note the low satisfaction for Chinese students reported in the 1993 paper). Postdocs are a highly international group and nationality has been found to be relevant to satisfaction (Sabharwal & Corley 2009).

 Figure 1 seemed especially problematic in terms of scale interpretation. This measurement seems to be on a different scale entirely, as it has a category >7, which would be impossible on a 1-7 scale.

 The authors should review literature about and applications of the SWLS to relevant populations more thoroughly and contextualize their findings in clear, comparable terms.

 Overall I did not find support for the claim that this survey showed surprisingly low levels of wellbeing.

 I’d like to conclude with a few points of agreement with prior research. For example, we also found that satisfaction does typically decline the longer someone is a postdoc (Miller & Feldman 2015).

 It is also not surprising that people lost interest in pursuing tenure-track academic appointments. If they did not lose interest, the competition for the relatively few available positions would be even more fierce, with perhaps even more stark disappointment among those not selected. Yet, while this competition has intensified, it is by no means new. Like entertainment and athletics, scientific careers function as tournaments (Freeman *et al. *2001). I agree with the authors’ recommendation that doctoral students and postdocs would benefit from more awareness of the structure of the scientific labor market.

 Prior research also supports the focus on improving the postdoc experience through interpersonal, lab-level interventions, rather than more tangible issues of pay and benefits (Miller & Feldman 2015; Scaffidi & Berman, 2011; Davis 2009). Research on postdoc satisfaction tends to identify the mentoring relationship and professional development as key to postdocs’ satisfaction.

 In summary, I have some fairly strong methodological reservations about this paper. As presented, the findings of dissatisfaction do not seem particularly strong. There are reasons to question the role of sample bias in selecting for more dissatisfied postdocs. However, I could support publication of a revised version that better contextualizes the paper with existing research, acknowledges the activist role of postdoc associations and resulting potential for bias, better examines the potential for nonresponse bias and missing data, and provides careful comparisons with other studies using the SWLS.

 Minor points

1.  For policy-relevant research, it is useful to report the time frame during which the data were collected.
2. Supplementary File 1 is described as having been distributed among 190 postdocs. In fact, it was completed by 190 postdocs and it is unknown to how many the survey was distributed. The NSF Survey of Graduate Students and Postdoctorates in Science and Engineering could provide an estimate of the number of postdocs at these institutions.
3. I do not usually use SPSS, so was not able to examine the data file. For what is probably a fairly manageable dataset like this, it would be helpful to provide a flat text file in a format like .csv.
4. Both supplementary files appear to link to the survey, not the list of postdoc associations.
5. Paragraph 1, “warranted” seems like an odd word choice here.

I have read this submission. I believe that I have an appropriate level of expertise to confirm that it is of an acceptable scientific standard, however I have significant reservations, as outlined above.

## References

[1] Davis.G: Improving the postdoctoral experience: An empirical approach. In Science and engineering careers in the United States: An analysis of markets and employment. *University of Chicago Press*.2009;99-127 Reference source

[2] FreemanRWeinsteinEMarincolaERosenbaumJSolomonF: Competition and careers in biosciences. *Science*.2001;294(5550) : 10.1126/science.1067477 2293-2294 10.1126/science.1067477 11743184

[3] MillerJ.MFeldmanM.P: isolated in the Lab: examining Dissatisfaction with Postdoctoral Appointments. *The Journal of Higher Education*.2015;86(5) :697-724 10.1080/00221546.2015.11777380

[4] SabharwalM: Faculty job satisfaction across gender and discipline. *The Social Science Journal*.2009;46(3) :539-556 10.1016/j.soscij.2009.04.015

[5] ScaffidiA.K: A positive postdoctoral experience is related to quality supervision and career mentoring, collaborations, networking and a nurturing research environment. *Higher Education*.2011;62(6) :685 Reference source

[6] PavotW: Review of the satisfaction with life scale. *Psychological assessment*.1993;5(2) :164 10.1037/1040-3590.5.2.164

[7] PavotW: The satisfaction with life scale and the emerging construct of life satisfaction. *The Journal of Positive Psychology*.2008;3(2) :137-152 10.1080/17439760701756946

# Response

**Published:** 2018-04-25

**Authors:** Amir Grinstein

**Affiliations:** Northeastern University & VU Amsterdam, USA

**Referee 1**

**Jennifer M. Miller**, USC Sol Price School of Public Policy, University of Southern California, Los Angeles, CA, USA 

**Approved with Reservations**

 This review will focus a critical eye on three issues, integration with existing literature, survey methodology, and use of the Satisfaction with Life Scale (SWLS). The review then acknowledges some points of agreement and support for the authors and ultimately recommends significant revision for publication, with some reservations.

 The abstract begins with the implication that the public discourse about postdocs is not informed by empirical work. The implication that empirical work is missing is a red flag to me *whenever* I encounter it, not only when it is my own empirical work that is being overlooked. Yet I do believe that our paper (Miller & Feldman, 2015) that uses the Science and Engineering PhD and Postdoc Survey (SEPPS) conducted by Sauermann and Roach to study dissatisfaction with postdoc appointments could have informed the design and interpretation of this study. So at the risk of being a caricature of reviewers who plug their own work, I will begin by pointing the authors toward this paper, which also contains a summary of prior research on postdoc satisfaction. The Scaffidi and Berman (2011) paper is also very relevant to the topic.

 I was pleased to see that the authors had cited Davis’s 2009 chapter analyzing the Sigma Xi survey of postdocs, as this has been some of the most influential empirical work on the postdoc experience. As a methodological step to avoid this type of disconnect from prior literature, I would recommend the practice of using Google Scholar’s “cited by” feature to look at the studies that have cited this chapter. It will connect the authors to even more recent and relevant empirical work.

 Further review of existing literature would also have been helpful to the authors in their use of the SWLS. Diener and colleagues have published a number of subsequent review articles (including but not limited to Diener & Pavot 1993 and Pavot & Diener 2008), but this study relies on the original 1985 paper.

**Reply: Thank you very much for directing us to highly relevant literature that we have overlooked. This is indeed an emerging and evolving area and we have now extended our literature search based on your guidance as well as integrated the above work into the revised manuscript.**

 The review will now turn to issues of interpretation related to the survey data and measurement scales.

 Interpretation of the survey data was complicated by the absence of a correlation table. It was not entirely clear, but it appears that conclusions are drawn from correlations, rather than regression analyses that more carefully model the relationship to satisfaction by including control variables. I recommend the authors clearly explain the analyses performed and provide a correlation table.

**Reply: We now include a correlation matrix. We also ran regression analysis on which we report in the revised version.**

 I have two concerns regarding the representativeness of the sample. First, the authors say the survey was distributed by postdoc associations *to their members*. Postdoc associations do not necessarily include all postdocs at an institution and often serve as agents of change, sometimes including formal unionization efforts. This is a limitation that would discourage me from drawing policy conclusions from the study. Postdoc association membership lists may overrepresent dissatisfied postdocs. Of course, it is also possible that at least some postdoc associations have a broader mailing list. It is even possible that not all postdoc associations distributed the survey at all. With only 29 associations, it seems likely it would be feasible to contact them to confirm distribution and to give some consideration to their mission statements.

 Second, in analyzing survey data, it is a questionable choice to include only complete cases. Various techniques for imputation allow the researchers to avoid discarding the valuable data from incomplete cases. The fact that age and gender were missing from over 20% of cases, while other variables were provided more completely, suggests that there may be some bias introduced by discarding missing data. Response bias also tends to overrepresent those with strong feelings and opinions.

 The likelihood for sample bias due to the reliance on postdoc associations is my main concern with the study as a whole. This cannot be remedied but can be acknowledged as an important limitation.

**Reply: We have decided to focus on postdoc associations given they represent many postdocs and enabled us one access point to large number of potential survey participants. Following your concern we now acknowledge this potential limitation in our GD.**

**About the missing data of gender and age: first, it seems reasonable to assume that missing data is equally distributed across the different postdoc associations and disciplines. Further, as these variables are only control variables rather than at the heart of our conceptualization we hope this limitation is not critical. Finally, it is important for us to clarify that we did use all the data available (we didn’t “throw” participant if s/he missed one of the questions).**

 Comparison of postdocs’ SWLS scores with early studies of relevant populations was complicated by the fact that the authors report the mean (4.47 on a 1-7 scale), while apparently other studies tend to report the total scale score (5-35). On page 2, the authors refer to 4 as the median, when 4 is more accurately described as the midpoint of the scale. Diener & Pavot (1993) are careful to interpret findings relevant to their scale anchors. A mean of 4.47 indicates a neutral to slightly satisfied group.

 But taking into account confidence intervals, it is not clear to me that the satisfaction of postdocs is significantly worse than that found by Diener & Pavot in 1993 for several groups of US college students. Given the cultural variation in reporting life satisfaction, the absence of nationality data also complicates interpretation of the scale (note the low satisfaction for Chinese students reported in the 1993 paper). Postdocs are a highly international group and nationality has been found to be relevant to satisfaction (Sabharwal & Corley 2009).

 Figure 1 seemed especially problematic in terms of scale interpretation. This measurement seems to be on a different scale entirely, as it has a category >7, which would be impossible on a 1-7 scale.

 The authors should review literature about and applications of the SWLS to relevant populations more thoroughly and contextualize their findings in clear, comparable terms.

 Overall I did not find support for the claim that this survey showed surprisingly low levels of wellbeing.

**Reply: This is a valuable discussion to have. Thank you. First, we used the 1-7 scale to be able to compare our results to some of the prior studies that did report their results on a 1-7 scale (e.g.,** **Kjeldstadli *et al.*, 2006;** **Samaranayake and Fernando, 2011).**

**While locating the 4.47 mean on the 5-35 SWLS scale suggests that our score falls under the “slightly satisfied” category (score = 22.35;** **Diener and Pavot, 1993** **) the finding is the lowest compared to all other student groups in developed countries or professionals (apart from “elderly caregivers”) as can be seen from Table 1 in** **Diener and Pavot (1993). According to these authors, satisfaction with life in the developed world is likely to hit “slightly satisfied” as a starting point. In this context our results actually show a surprisingly low satisfaction with life of postdocs. We now integrate this discussion in the text.**

**Your point about the diverse cultures and countries postdocs come from and how this plays a role is a very valid one and we are now discussing this as an avenue for future research.**

**We now use the term “midpoint” rather than “median” when addressing the score 4.**

**The decision to adapt our scale for the purpose of the figure appeared in footnote 3 in the original version of the manuscript: “** **To be compatible with Abdallah *et al.*’s (2009) map, we transformed our 1-7 satisfaction with life scale to a 1-10 scale, on which the surveyed postdocs demonstrate a 6.1 satisfaction with life score.” Still, following the feedback from the review team we have decided to remove Figure 1 from the revised version.**

****

 I’d like to conclude with a few points of agreement with prior research. For example, we also found that satisfaction does typically decline the longer someone is a postdoc (Miller & Feldman 2015).

 It is also not surprising that people lost interest in pursuing tenure-track academic appointments. If they did not lose interest, the competition for the relatively few available positions would be even more fierce, with perhaps even more stark disappointment among those not selected. Yet, while this competition has intensified, it is by no means new. Like entertainment and athletics, scientific careers function as tournaments (Freeman *et al. *2001). I agree with the authors’ recommendation that doctoral students and postdocs would benefit from more awareness of the structure of the scientific labor market.

 Prior research also supports the focus on improving the postdoc experience through interpersonal, lab-level interventions, rather than more tangible issues of pay and benefits (Miller & Feldman 2015; Scaffidi & Berman, 2011; Davis 2009). Research on postdoc satisfaction tends to identify the mentoring relationship and professional development as key to postdocs’ satisfaction.

**Reply: These are important points and highly relevant sources. We connect them now with our findings and implications.**

 In summary, I have some fairly strong methodological reservations about this paper. As presented, the findings of dissatisfaction do not seem particularly strong. There are reasons to question the role of sample bias in selecting for more dissatisfied postdocs. However, I could support publication of a revised version that better contextualizes the paper with existing research, acknowledges the activist role of postdoc associations and resulting potential for bias, better examines the potential for nonresponse bias and missing data, and provides careful comparisons with other studies using the SWLS.

**Reply: Thank you. We have made an attempt to address all your comments and benefit significantly from your valuable feedback.**

 Minor points

1.  For policy-relevant research, it is useful to report the time frame during which the data were collected.

**Reply: We now add the time frame of the study.  **

1. Supplementary File 1 is described as having been distributed among 190 postdocs. In fact, it was completed by 190 postdocs and it is unknown to how many the survey was distributed. The NSF Survey of Graduate Students and Postdoctorates in Science and Engineering could provide an estimate of the number of postdocs at these institutions.

**Reply: First, we change the wording to “completed by”.**

**Also, we now refer to the overall sample frame of postdocs in footnote 1.**

 I do not usually use SPSS, so was not able to examine the data file. For what is probably a fairly manageable dataset like this, it would be helpful to provide a flat text file in a format like .csv.

**Reply: We have now submitted both SPSS and Excel files.  **

1. Both supplementary files appear to link to the survey, not the list of postdoc associations.

**Reply: We made sure this is corrected. **

1. Paragraph 1, “warranted” seems like an odd word choice here.

**Reply: We have modified the sentence.**

**References**

 1. Davis.G: Improving the postdoctoral experience: An empirical approach. In Science and engineering careers in the United States: An analysis of markets and employment. *University of Chicago Press*. 2009. 99-127 Reference Source 

 2. Freeman R, Weinstein E, Marincola E, Rosenbaum J, Solomon F: Competition and careers in biosciences. *Science*. 2001; **294** (5550): 2293-2294 Publisher Full Text 

 3. Miller J.M, Feldman M.P: isolated in the Lab: examining Dissatisfaction with Postdoctoral Appointments. *The Journal of Higher Education*. 2015; **86** (5): 697-724 Reference Source 

 4. Sabharwal M: Faculty job satisfaction across gender and discipline. *The Social Science Journal*. 2009; **46** (3): 539-556 Reference Source 

 5. Scaffidi A.K: A positive postdoctoral experience is related to quality supervision and career mentoring, collaborations, networking and a nurturing research environment. *Higher Education*. 2011; **62** (6): 685 Reference Source 

 6. Pavot W: Review of the satisfaction with life scale. *Psychological assessment*. 1993; **5** (2): 164 Reference Source 

 7. Pavot W: The satisfaction with life scale and the emerging construct of life satisfaction. *The Journal of Positive Psychology*. 2008; **3** (2): 137-152 Reference Source

# Peer review: Referee response for version 1

**DOI:** 10.5256/f1000research.13576.r26673

**Published:** 2017-10-12

**Authors:** Gary S. McDowell

**Affiliations:** 1 The Future of Research Inc., 848 Brockton Avenue, Abington, MA, USA

In “The Unhappy Postdoc”, Grinstein and Treister present the results of a survey to 190 respondents who carried out postdoctoral training in the U.S. The results seek to demonstrate the satisfaction of postdocs with their postdoctoral training and isolate factors which are critical to their sense of well-being. So far, very few efforts have looked at the well-being of postdocs in the context of their training, and this survey data sheds some light into the murky world of the postdoc.

 The survey has a small sample size, understandable given the difficulty of actually getting access to communicate with postdocs, but the most pressing concerns are how far selection bias plays into the inclination of postdocs to respond; and whether there was thought given to an appropriate control group? However, it is encouraging to see that some of the results replicate the findings of others, which may hint towards this being at least a fair representation of a subset of the postdoc population.

 Major concerns:

- Please check the proportion of US PhDs pursuing postdocs - while this is not an easy number to quantify, Kahn and Ginther 2017 ^1^ and Sauermann and Roach 2016 ^2^ both point to about 80% of U.S. biomedical PhDs pursuing postdocs, and the NSF’s Survey of Earned Doctorates suggests that at graduation, 30% of PhDs have a postdoc lined up, and 50% don’t know what they are doing next, suggesting a large number of people are defaulting into this plan. Again also check the tenure-track position percentage from more recent literature/qualify - it may be as low as 8% for tenure-track positions (although this may be for research-intensive institutions specifically).
- Sauermann and Roach 2016 ^2^ should be cited - theirs is a larger survey effort of postdocs that addresses some of the same issues, particularly the change in career preference over time, and comparison with Figure 2 would be particularly useful.
- The first sentence in the results section would make more sense being placed after the sentence that follows it, which lays out the scale. Also the authors should explain why a mean of 4.47 and 30% of postdocs lying below the median supports their argument - what is the mean expected from other studies using the same scale? This is suggested, and one example is given, but more examples and clearer articulation of the comparison would be helpful. Also, if 30% are below the median, does that not mean 70% are at or above? It is not clear why this result is indicative of poor satisfaction and the authors should make this clearer.
- How does selection bias factor into the author’s analysis? One counter-argument is that the people with most to complain about felt most compelled to reply, and this skewed the results - how did the authors account for this, or is it possible to do so?
- Could the authors comment further on the redirection of postdocs to industry or to other non-academic careers, reflecting on the state of the current labor market? The 2012 NIH Biomedical Workforce Working Group Report found that industry is actually hiring fewer people into research positions, and into more managerial/non-research positions, and our own work looking at Census data ^3^ show that the private sector is actually a shrinking component of the biomedical workforce, apparently explained by the increasing outsourcing of R&D to academic labs, who are both increasingly desperate for sources of funding, and also provide a plentiful pool of cheap labor. Please see also Mason *et al*., 2016 ^4^.
- Reference could also be made to Faupel-Badger *et al*., 2017 ^5^, which is possibly the only other citation that looks at career satisfaction from postdoctoral training.
- Can the authors link their findings to recent work that suggests that it is not the labor market, but the nature of the academic position itself, which is driving researchers out of academia? See Sauermann and Roach 2017 ^6^.
- In Table 1, what does 'number of papers' show - is it the number published during a postdoc? In total? The survey asks this, but the table doesn't make clear which data this is. Both numbers would actually be very interesting, and also if it's possible to show how many papers people had from their first postdoc before possibly doing another one.

Minor concerns:

- “Their research requires heavier funding” - could the authors articulate what they mean here? Is it that research generally has become more expensive? Or is there some increased burden at the postdoctoral position?
- The increasing cost of research and the market forces that are resulting in more postdocs (that they are essentially cheaper than graduate students) have been pointed out by Paula Stephan in her book “How Economics Shapes Science” ^7^ and elsewhere, and these works should be cited appropriately in the introduction
- “Moreover, postdocs that are not able to achieve an academic appointment often become over-qualified for industry positions while losing alternative higher compensation (salaries in the industry)” - Kahn and Ginther 2017 ^1^ should be cited here.
- Footnote 3 should probably be moved into the Figure Legend of Figure 1, to highlight transformation of the 1-7 scale into a 1-10 scale.
- In Table 1, “Duration of postdoc (years)” may be better described as “Total years in postdoctoral positions” or similar, if the survey questions are understood correctly - it is not clear whether this is pointing out the years in the current postdoc, or the years of postdoccing in total.
- Supplementary File 2 is not as described, but is the same as Supplementary File 1.

I have read this submission. I believe that I have an appropriate level of expertise to confirm that it is of an acceptable scientific standard, however I have significant reservations, as outlined above.

## References

[1] KahnSGintherDK: The impact of postdoctoral training on early careers in biomedicine. *Nat Biotechnol*.2017;35(1) : 10.1038/nbt.3766 90-94 10.1038/nbt.3766 28072769

[2] SauermannHRoachM: SCIENTIFIC WORKFORCE. Why pursue the postdoc path?. *Science*.2016;352(6286) : 10.1126/science.aaf2061 663-4 10.1126/science.aaf2061 27151854

[3] HeggenessML: Preparing for the 21st Century Biomedical Research Job Market: Using Census Data to Inform Policy and Career Decision-Making. *Self-Journals of Science*.2016;

[4] MasonJLJohnstonEBerndtSSegalKLeiMWiestJS: Labor and skills gap analysis of the biomedical research workforce. *FASEB J*.2016;30(8) : 10.1096/fj.201500067R 2673-83 10.1096/fj.201500067R 27075242PMC4970602

[5] Faupel-BadgerJMNelsonDEIzmirlianG: Career Satisfaction and Perceived Salary Competitiveness among Individuals Who Completed Postdoctoral Research Training in Cancer Prevention. *PLoS One*.2017;12(1) : 10.1371/journal.pone.0169859 e0169859 10.1371/journal.pone.0169859 28121985PMC5266270

[6] RoachMSauermannH: The declining interest in an academic career. *PLoS One*.2017;12(9) : 10.1371/journal.pone.0184130 e0184130 10.1371/journal.pone.0184130 28922403PMC5602526

[7] StephanP: How Economics Shapes Science. *Harvard University Press, Cambridge, MA, USA*.2012;

# Response

**Published:** 2018-04-25

**Authors:** Amir Grinstein

**Affiliations:** Northeastern University & VU Amsterdam, USA

**Referee 2**

**Gary S McDowell**, The Future of Research Inc., 848 Brockton Avenue, Abington, MA, USA;  Manylabs, 1086 Folsom Street, San Francisco, CA, USA 

**Approved with Reservations**

 In “The Unhappy Postdoc”, Grinstein and Treister present the results of a survey to 190 respondents who carried out postdoctoral training in the U.S. The results seek to demonstrate the satisfaction of postdocs with their postdoctoral training and isolate factors which are critical to their sense of well-being. So far, very few efforts have looked at the well-being of postdocs in the context of their training, and this survey data sheds some light into the murky world of the postdoc.

 The survey has a small sample size, understandable given the difficulty of actually getting access to communicate with postdocs, but the most pressing concerns are how far selection bias plays into the inclination of postdocs to respond; and whether there was thought given to an appropriate control group? However, it is encouraging to see that some of the results replicate the findings of others, which may hint towards this being at least a fair representation of a subset of the postdoc population.

 Major concerns:

- Please check the proportion of US PhDs pursuing postdocs - while this is not an easy number to quantify, Kahn and Ginther 2017 ^1^ and Sauermann and Roach 2016 ^2^ both point to about 80% of U.S. biomedical PhDs pursuing postdocs, and the NSF’s Survey of Earned Doctorates suggests that at graduation, 30% of PhDs have a postdoc lined up, and 50% don’t know what they are doing next, suggesting a large number of people are defaulting into this plan. Again also check the tenure-track position percentage from more recent literature/qualify - it may be as low as 8% for tenure-track positions (although this may be for research-intensive institutions specifically).

**Reply: Thank you for these valuable sources. Based on these suggestions we updated our introduction to the manuscript. **

- Sauermann and Roach 2016 ^2^ should be cited - theirs is a larger survey effort of postdocs that addresses some of the same issues, particularly the change in career preference over time, and comparison with Figure 2 would be particularly useful.

**Reply: Thank you for suggesting this. This paper - and other sources suggested by the review team - are now cited and integrated into our discussion of the dynamics of postdocs’ career path.  **

- The first sentence in the results section would make more sense being placed after the sentence that follows it, which lays out the scale. Also the authors should explain why a mean of 4.47 and 30% of postdocs lying below the median supports their argument - what is the mean expected from other studies using the same scale? This is suggested, and one example is given, but more examples and clearer articulation of the comparison would be helpful. Also, if 30% are below the median, does that not mean 70% are at or above? It is not clear why this result is indicative of poor satisfaction and the authors should make this clearer.

**Reply: First, we made the suggested editing change. Also, w** **e now present our results more clearly in the context of other studies examining various populations’ life satisfaction (a good review is** **Diener and Pavot, 1993** **). The mean life satisfaction we found for postdocs is the lowest compared to all other student groups in developed countries or professionals (apart from “elderly caregivers”) as can be seen from Table 1 in** **Diener and Pavot (1993). **

- How does selection bias factor into the author’s analysis? One counter-argument is that the people with most to complain about felt most compelled to reply, and this skewed the results - how did the authors account for this, or is it possible to do so?

**Reply: This is a valid point. One way of addressing this is indeed comparing our findings to many other populations and studies – as we do now more rigorously do – with the understanding that such a potential bias can play in any surveyed group. Further, we now acknowledge this potential limitation in our GD.**

- Could the authors comment further on the redirection of postdocs to industry or to other non-academic careers, reflecting on the state of the current labor market? The 2012 NIH Biomedical Workforce Working Group Report found that industry is actually hiring fewer people into research positions, and into more managerial/non-research positions, and our own work looking at Census data ^3^ show that the private sector is actually a shrinking component of the biomedical workforce, apparently explained by the increasing outsourcing of R&D to academic labs, who are both increasingly desperate for sources of funding, and also provide a plentiful pool of cheap labor. Please see also Mason *et al*., 2016 ^4^.

**Reply: Thank you for highlighting these evidence and research. We now integrate them to our discussion of the study’s implications. **

- Reference could also be made to Faupel-Badger *et al*., 2017 ^5^, which is possibly the only other citation that looks at career satisfaction from postdoctoral training.

**Reply: Thank you. We now cite this work. **

- Can the authors link their findings to recent work that suggests that it is not the labor market, but the nature of the academic position itself, which is driving researchers out of academia? See Sauermann and Roach 2017 ^6^.

**Reply: We now include this idea in our concluding discussion.  **

- In Table 1, what does 'number of papers' show - is it the number published during a postdoc? In total? The survey asks this, but the table doesn't make clear which data this is. Both numbers would actually be very interesting, and also if it's possible to show how many papers people had from their first postdoc before possibly doing another one.

**Reply: Currently we reported on the total number of publications. We now improve the labeling and add a column for number publications during the postdoc. Unfortunately, we do not have the data about publications per postdoc fellowship.**

 Minor concerns:

- “Their research requires heavier funding” - could the authors articulate what they mean here? Is it that research generally has become more expensive? Or is there some increased burden at the postdoctoral position?

**Reply: This refers to the burden of maintaining a lab and conducting top-quality scientific work. We are now more explicit in the text and add a supporting citation.  **

- The increasing cost of research and the market forces that are resulting in more postdocs (that they are essentially cheaper than graduate students) have been pointed out by Paula Stephan in her book “How Economics Shapes Science” ^7^ and elsewhere, and these works should be cited appropriately in the introduction

**Reply: Thank you for this valuable source. We now included it in the introduction.**

- “Moreover, postdocs that are not able to achieve an academic appointment often become over-qualified for industry positions while losing alternative higher compensation (salaries in the industry)” - Kahn and Ginther 2017 ^1^should be cited here.

**Reply: Thank you!**

- Footnote 3 should probably be moved into the Figure Legend of Figure 1, to highlight transformation of the 1-7 scale into a 1-10 scale.

**Reply: Given reviewers’ feedback we have decided to remove Figure 1.**

- In Table 1, “Duration of postdoc (years)” may be better described as “Total years in postdoctoral positions” or similar, if the survey questions are understood correctly - it is not clear whether this is pointing out the years in the current postdoc, or the years of postdoccing in total.

**Reply: Done.**

- Supplementary File 2 is not as described, but is the same as Supplementary File 1.

**Reply: We now corrected this. Thank you.**

**References**

 1. Kahn S, Ginther DK: The impact of postdoctoral training on early careers in biomedicine. *Nat Biotechnol*. 2017; **35** (1): 90-94 PubMed Abstract | Publisher Full Text 

 2. Sauermann H, Roach M: SCIENTIFIC WORKFORCE. Why pursue the postdoc path?. *Science*. 2016; **352** (6286): 663-4 PubMed Abstract | Publisher Full Text 

 3. Heggeness ML: Preparing for the 21st Century Biomedical Research Job Market: Using Census Data to Inform Policy and Career Decision-Making. *Self-Journals of Science*. 2016. Publisher Full Text 

 4. Mason JL, Johnston E, Berndt S, Segal K, Lei M, Wiest JS: Labor and skills gap analysis of the biomedical research workforce. *FASEB J*. 2016; **30** (8): 2673-83 PubMed Abstract | Publisher Full Text 

 5. Faupel-Badger JM, Nelson DE, Izmirlian G: Career Satisfaction and Perceived Salary Competitiveness among Individuals Who Completed Postdoctoral Research Training in Cancer Prevention. *PLoS One*. 2017; **12** (1): e0169859 PubMed Abstract| Publisher Full Text 

 6. Roach M, Sauermann H: The declining interest in an academic career. *PLoS One*. 2017; **12** (9): e0184130 PubMed Abstract | Publisher Full Text 

 7. Stephan P: How Economics Shapes Science. *Harvard University Press, Cambridge, MA, USA*. 2012.

# Peer review: Referee response for version 1

**DOI:** 10.5256/f1000research.13576.r26676

**Published:** 2017-10-11

**Authors:** Nick Riddiford

**Affiliations:** 1 Institut Curie, Paris, France

Grinstein and Treister present and discuss survey data collected from 190 US-based postdoctoral researchers examining life satisfaction and career goals of respondents. Their data suggest that postdocs in the US are less satisfied with their life than the general population, and that their career goals drift further away from academic research as they progress though their fellowship(s). Such data is useful and adds to a growing body of evidence that point to systemic failures in the way postdocs are treated.

 While Grinstein and Treister do a good job of describing the data, the analysis is fairly light, and several questions are left unconsidered. Most importantly, in looking for correlates of life satisfaction, what does it say that the explanatory variables you have used don’t explain this data? In addition to several major revisions, I also suggest that the authors discuss how their work relates to similar data from recent articles (for example [ref1] ^-^^4^.

**Major revisions**

1. Considering the general tone of the manuscript is descriptive, I would strongly urge plotting each of the correlation analyses (life satisfaction vs gender, age …), and reporting R ^2^ values (I think that these are easily generated as the tests have already been done) and simply discussing any observed correlations. The lack of significance in such a small dataset does not automatically indicate that there is nothing interesting going on. At the very least, I think the correlation between “lab atmosphere” and life satisfaction should be plotted, and the tests/software used for the correlation analyses clearly detailed in the methods section (linear regression?). Furthermore, a correlation coefficient of 0.247 is reported  – is this really the R value or the R ^2^? If the former, then the R ^2^ = 0.06, which means that “lab atmosphere” explains only 6% of the variability in reported life satisfaction scores, which suggests there could be some other explanatory variables that haven’t been considered. This should be discussed.  
2. A second major issue relates to Figure 1. Here, it is reported in the first sentence of the results that the mean satisfaction score was 4.47, but the colouring of figure 1 suggests that postdocs fall in the 5.5-7.0 bracket. In either case, I would suggest that this only serves to highlight the many caveats of a) self-reporting of life satisfaction and b) making comparisons between data sets collected under different conditions. Do the authors really think that, with a mean life satisfaction of 4.47, postdocs in the US are (quite considerably) less satisfied with life than residents of some of the poorest, most poverty-stricken countries in the world? If not, I recommend that this figure be removed entirely. Perhaps a more meaningful comparison to make would be between the US population (per state/demographic?) and US-based postdocs.
3. To ensure reproducibility, Dataset 1 should be resubmitted as an excel compatible file (or.txt, .csv) instead of, or in addition to, the .sav they currently provide. 

**Minor points**

1. What do the alpha values refer to throughout the manuscript (e.g. α=.90)? These either need to be removed or discussed in the Methods section.
2. Table 1 should be more formally presented (or see suggestion 1) and formatted i.e succinct, descriptive title and footnote describing ‘n’, and why there are missing values i.e discrepancy between 190 and 178). This applies to all other figures: The title should be concise, and followed by a legend that describes the figure in sufficient detail. Commentaries on the figure – such as in Fig.2 – belong in the main body of the results section.
3. Why do you make the assumption that postdocs who answered your survey are “typically already affiliated with a top-tier academic affiliation”?
4. “Satisfaction with life” should be replaced with “life satisfaction” throughout manuscript.
5. Remove “Less than a third” in sentence containing “Only 28.4% “agreed” or “definitely agreed” to recommend the postdoc path to others. Less than a third.”.
6. Figure 2 should be reproduced at a higher quality – it appears very pixilated on my screen.

 

**Grammatical mistakes**

 Introduction

- Replace “kids” with “children” or similar in sentence containing “personal life (marriage, kids) on hold.”

Results

- Replace “Further” with “Furthermore” in sentence containing “Further, 30% of participants demonstrate”
- Consider revising repetitive sentence containing “typically already affiliated with a top-tier academic affiliation”
- Pluralise “type” in sentence containing “Further, prior work on satisfaction with life of other type of skilled trainees”

Discussion

- Replace “scarce” with “scarcer” or “more scarce” in sentence containing “On the other hand, however, the growing realization that these positions are scarce than ever before”
- Remove “they” from sentence containing “they should consider adopting”
- Catalysts should not be plural in sentence containing “could be another catalysts for postdocs to re-think”

 

**Several recent survey-based articles that would be worth comparing to**

- Reader’s responses from Nature poll ^1^
- Analysis of academic workforce in US ^3^
- Large analysis of US census data ^2^
- Survey of UK-based biomedical researchers ^4^
- The Royal Society: The Scientific Century: securing our future prosperity. 2010

 

**Suggestions**

- Table 1 would be better represented as a several bar charts
- You report that “out of a list of potential explanatory variables of satisfaction with life among postdocs … only one factor …”. Even if this is mostly negative data, this would make a nice figure, as it’s quite surprising to me that you find no correlation between some of your explanatory variables
- Representing Fig.2 as a 3D bar chart adds nothing at a loss of clarity. Consider replotting as a conventional 2D bar chart  

I have read this submission. I believe that I have an appropriate level of expertise to confirm that it is of an acceptable scientific standard, however I have significant reservations, as outlined above.

## References

[1] PowellK: Hard work, little reward: Nature readers reveal working hours and research challenges. *Nature*.2016; 10.1038/nature.2016.20933 10.1038/nature.2016.20933

[2] HeggenessMLGunsalusKTPacasJMcDowellGS: Preparing for the 21st Century Biomedical Research Job Market: Using Census Data to Inform Policy and Career Decision-Making. *sjscience.org*.2016;

[3] HeggenessMLGunsalusKTWPacasJMcDowellG: Snapshot of the US biomedical workforce. *Nature*.2016;541:21-23

[4] RiddifordN: A survey of working conditions within biomedical research in the United Kingdom. *F1000Research*.2017;6: 10.12688/f1000research.11029.1 10.12688/f1000research.11029.1 29043063PMC5627577

# Response

**Published:** 2018-04-25

**Authors:** Amir Grinstein

**Affiliations:** Northeastern University & VU Amsterdam, USA

**Referee 3**

**Nick Riddiford**, Institut Curie, Paris, France 

**Approved with Reservations**

 Grinstein and Treister present and discuss survey data collected from 190 US-based postdoctoral researchers examining life satisfaction and career goals of respondents. Their data suggest that postdocs in the US are less satisfied with their life than the general population, and that their career goals drift further away from academic research as they progress though their fellowship(s). Such data is useful and adds to a growing body of evidence that point to systemic failures in the way postdocs are treated.

 While Grinstein and Treister do a good job of describing the data, the analysis is fairly light, and several questions are left unconsidered. Most importantly, in looking for correlates of life satisfaction, what does it say that the explanatory variables you have used don’t explain this data? In addition to several major revisions, I also suggest that the authors discuss how their work relates to similar data from recent articles (for example [ref1] ^-^ ^4^.

**Major revisions**

1. Considering the general tone of the manuscript is descriptive, I would strongly urge plotting each of the correlation analyses (life satisfaction vs gender, age …), and reporting R ^2^ values (I think that these are easily generated as the tests have already been done) and simply discussing any observed correlations. The lack of significance in such a small dataset does not automatically indicate that there is nothing interesting going on. At the very least, I think the correlation between “lab atmosphere” and life satisfaction should be plotted, and the tests/software used for the correlation analyses clearly detailed in the methods section (linear regression?). Furthermore, a correlation coefficient of 0.247 is reported – is this really the R value or the R ^2^? If the former, then the R ^2^ = 0.06, which means that “lab atmosphere” explains only 6% of the variability in reported life satisfaction scores, which suggests there could be some other explanatory variables that haven’t been considered. This should be discussed.  

**Reply: Thank you for pushing us to more rigorously test our results. We now report in more detail the correlation matrix and a more rigorous regression analysis. **

1. A second major issue relates to Figure 1. Here, it is reported in the first sentence of the results that the mean satisfaction score was 4.47, but the colouring of figure 1 suggests that postdocs fall in the 5.5-7.0 bracket. In either case, I would suggest that this only serves to highlight the many caveats of a) self-reporting of life satisfaction and b) making comparisons between data sets collected under different conditions. Do the authors really think that, with a mean life satisfaction of 4.47, postdocs in the US are (quite considerably) less satisfied with life than residents of some of the poorest, most poverty-stricken countries in the world? If not, I recommend that this figure be removed entirely. Perhaps a more meaningful comparison to make would be between the US population (per state/demographic?) and US-based postdocs.

**Reply:** **We now present our results more clearly in the context of other studies examining various populations’ life satisfaction (a good review is** **Diener and Pavot, 1993** **). The mean life satisfaction we found for postdocs is the lowest compared to all other student groups in developed countries or professionals (apart from “elderly caregivers”) as can be seen from Table 1 in** **Diener and Pavot (1993).**

**We are sorry for not being clear enough about the difference between the text and the use of our scale and the Figure. The decision to adapt our scale for the purpose of the figure appeared in footnote 3 of the original version of the manuscript: “** **To be compatible with Abdallah *et al.*’s (2009) map, we transformed our 1-7 satisfaction with life scale to a 1-10 scale, on which the surveyed postdocs demonstrate a 6.1 satisfaction with life score.” Still, given the feedback from the review team we have decided to remove Figure 1 from the revised manuscript.**

**Further, following your suggestions we toned down our discussion and removed some of the “not clean” comparisons. Noteworthy that our findings do suggest that in the context of developed countries, our results actually show a surprisingly low life satisfaction score of postdocs. We now integrate this discussion in the text. **

1. To ensure reproducibility, Dataset 1 should be resubmitted as an excel compatible file (or.txt, .csv) instead of, or in addition to, the .sav they currently provide. 

**Reply: Done.**

 Minor points

1. What do the alpha values refer to throughout the manuscript (e.g. α=.90)? These either need to be removed or discussed in the Methods section.

**Reply: Sorry for not being clear. We now explain this better in the text (Cronbach’s Alpha that tests for the reliability of the scale).**

1. Table 1 should be more formally presented (or see suggestion 1) and formatted i.e succinct, descriptive title and footnote describing ‘n’, and why there are missing values i.e discrepancy between 190 and 178). This applies to all other figures: The title should be concise, and followed by a legend that describes the figure in sufficient detail. Commentaries on the figure – such as in Fig.2 – belong in the main body of the results section.

**Reply: We now include a correlation matrix in addition to the table describing participants’ profiles. We also added a table for the regression analysis results. We moved the elaborated text from Fig. 2. We kept (and better explained) some of the text below the tables that seemed the most relevant to interpret the results (coding of some variables, p values). **

1. Why do you make the assumption that postdocs who answered your survey are “typically already affiliated with a top-tier academic affiliation”?

**Reply: This is a fair point. We removed this unfounded assumption.  **

1. “Satisfaction with life” should be replaced with “life satisfaction” throughout manuscript.

**Reply: Our use of “satisfaction with life” was influenced by the fact that this is the way the established measure is labeled. In the revised version we kept this term only when addressing the scale and in all other locations changed to “life satisfaction”.  **

1. Remove “Less than a third” in sentence containing “Only 28.4% “agreed” or “definitely agreed” to recommend the postdoc path to others. Less than a third.”.

**Reply: Done.**

1. Figure 2 should be reproduced at a higher quality – it appears very pixilated on my screen.

- **Reply: We replotted this figure as 2D bar chart and changed the colors. We hope it is clearer now. Also, please note this is now Fig. 1.**

Grammatical mistakes

 Introduction

- Replace “kids” with “children” or similar in sentence containing “personal life (marriage, kids) on hold.”

**Reply: Done.**

 Results

- Replace “Further” with “Furthermore” in sentence containing “Further, 30% of participants demonstrate”

**Reply: Done.  **

- Consider revising repetitive sentence containing “typically already affiliated with a top-tier academic affiliation”
- Pluralise “type” in sentence containing “Further, prior work on satisfaction with life of other type of skilled trainees”

- Replace “scarce” with “scarcer” or “more scarce” in sentence containing “On the other hand, however, the growing realization that these positions are scarce than ever before”
- Remove “they” from sentence containing “they should consider adopting”
- Catalysts should not be plural in sentence containing “could be another catalysts for postdocs to re-think”

**Reply: We have corrected (or removed) the relevant sentences.**

 Several recent survey-based articles that would be worth comparing to

- Reader’s responses from Nature poll ^1^
- Analysis of academic workforce in US ^3^
- Large analysis of US census data ^2^
- Survey of UK-based biomedical researchers ^4^
- The Royal Society: The Scientific Century: securing our future prosperity. 2010

**Reply: Thank you. We have integrated some of the work that studied these sources throughout the revised manuscript.**

  Suggestions

- Table 1 would be better represented as a several bar charts

**Reply: As we are more used to the “standard” way of reporting participants’ characteristics in a table we thought most readers would prefer a “standard” table. If this is key, we can of course make the change in a next revision effort. **

- You report that “out of a list of potential explanatory variables of satisfaction with life among postdocs … only one factor …”. Even if this is mostly negative data, this would make a nice figure, as it’s quite surprising to me that you find no correlation between some of your explanatory variables

**Reply: We were not sure what type of figure would capture best this effect. In the revised version we also include a table for the regression analysis results which make results more clear and accessible. We are happy to create a figure if you have an idea about the most relevant approach. **

- Representing Fig.2 as a 3D bar chart adds nothing at a loss of clarity. Consider replotting as a conventional 2D bar chart  

**Reply: Done.  Please note this is Fig. 1 now.**

**References**

 1. Powell K: Hard work, little reward: Nature readers reveal working hours and research challenges. *Nature*. 2016. Publisher Full Text 

 2. Heggeness ML, Gunsalus KT, Pacas J, McDowell GS: Preparing for the 21st Century Biomedical Research Job Market: Using Census Data to Inform Policy and Career Decision-Making. *sjscience.org*. 2016. 

 3.  Heggeness ML, Gunsalus KTW, Pacas J, McDowell G: Snapshot of the US biomedical workforce. *Nature*. 2016; **541**: 21-23

 4. Riddiford N: A survey of working conditions within biomedical research in the United Kingdom. *F1000Research*. 2017; **6**. Publisher Full Text

# Peer review: Referee response for version 1

**DOI:** 10.5256/f1000research.13576.r26232

**Published:** 2017-10-09

**Authors:** Navid Ghaffarzadegan, Ran Xu

**Affiliations:** 1 Grado Department of Industrial & Systems Engineering (ISE), Virginia Tech, Blacksburg, VA, USA; 2 Virginia Tech, Blacksburg, VA, USA

Overall this is an interesting work and it is well-written. The study explores satisfaction with life of postdocs. It also looks at career goal change of postdocs. The study contributes to our understanding of the current mental state of postdocs, a growing sub-population of the science workforce.

 The work is clearly and accurately presented. However, there are many other current literature on this topic that authors may benefit from reading them and linking their literature review to those studies or comparing and contrasting their findings.

 The study design is appropriate. There are two concerns: first, as the authors acknowledge the response rate was not available. Second, biomedical postdocs in this data seem to be over-represented when compared to the national sample in Powell (2015) ^1^. As reported in other studies ^2^^-^^3^, the chance of landing tenure-track positions for PhDs of biomedical sciences and engineering is relatively small, which may affect their satisfaction. 

 Most of the analysis are descriptive, which is fine. The interpretations are mostly accurate. The relationship between lab atmosphere and satisfaction is interesting. However, one might worried it’s confounded by individual characteristics such as personality – that is, people with more extraversion or agreeableness will score high on both satisfaction and subjective lab atmosphere. Furthermore, a more interesting/appropriate analysis for figure 2 might be to present results for subgroups by duration of the postdoc position.

 Most of the source data underlying the results are available to ensure full reproducibility. It seems that the link for Supplementary file 2 leads to the same source as for Supplementary file 1 which can be fixed.

 Finally, most of the conclusions are adequately supported by the results. The only problem was the suggestion of focusing on soft (non-science) factors of the lab. While the point makes sense, since the paper is descriptive, the authors should avoid causal interpretations.

We have read this submission. We believe that we have an appropriate level of expertise to confirm that it is of an acceptable scientific standard, however we have significant reservations, as outlined above.

## References

[1] PowellK: The future of the postdoc. *Nature*.2015;520(7546) : 10.1038/520144a 144-7 10.1038/520144a 25855437

[2] LarsonRCGhaffarzadeganNXueY: Too Many PhD Graduates or Too Few Academic Job Openings: The Basic Reproductive Number R0 in Academia. *Syst Res Behav Sci*.31(6) : 10.1002/sres.2210 745-750 10.1002/sres.2210 25642132PMC4309283

[3] GhaffarzadeganNHawleyJLarsonRXueY: A Note on PhD Population Growth in Biomedical Sciences. *Syst Res Behav Sci*.23(3) : 10.1002/sres.2324 402-405 10.1002/sres.2324 26190914PMC4503365

# Response

**Published:** 2018-04-25

**Authors:** Amir Grinstein

**Affiliations:** Northeastern University & VU Amsterdam, USA

**Referee 4**

**Navid Ghaffarzadegan**, Grado Department of Industrial & Systems Engineering (ISE), Virginia Tech, Blacksburg, VA, USA 

**Ran Xu**, Virginia Tech, Blacksburg, VA, USA 

**Approved with Reservations**

 Overall this is an interesting work and it is well-written. The study explores satisfaction with life of postdocs. It also looks at career goal change of postdocs. The study contributes to our understanding of the current mental state of postdocs, a growing sub-population of the science workforce.

 The work is clearly and accurately presented. However, there are many other current literature on this topic that authors may benefit from reading them and linking their literature review to those studies or comparing and contrasting their findings.

**Reply: Thank you for this suggestion. We have followed the review team recommendation and update our literature review.**

 The study design is appropriate. There are two concerns: first, as the authors acknowledge the response rate was not available. Second, biomedical postdocs in this data seem to be over-represented when compared to the national sample in Powell (2015) ^1^. As reported in other studies ^2^ ^-^ ^3^, the chance of landing tenure-track positions for PhDs of biomedical sciences and engineering is relatively small, which may affect their satisfaction. 

**Reply: Thank you for this feedback which we now acknowledge as a potential limitation of our work.**

 Most of the analysis are descriptive, which is fine. The interpretations are mostly accurate. The relationship between lab atmosphere and satisfaction is interesting. However, one might worried it’s confounded by individual characteristics such as personality – that is, people with more extraversion or agreeableness will score high on both satisfaction and subjective lab atmosphere. Furthermore, a more interesting/appropriate analysis for figure 2 might be to present results for subgroups by duration of the postdoc position.

**Reply: To account for multiple factors and provide a more rigorous testing of the data we now report a regression analysis.**

 Most of the source data underlying the results are available to ensure full reproducibility. It seems that the link for Supplementary file 2 leads to the same source as for Supplementary file 1 which can be fixed.

**Reply: We corrected for this.**

 Finally, most of the conclusions are adequately supported by the results. The only problem was the suggestion of focusing on soft (non-science) factors of the lab. While the point makes sense, since the paper is descriptive, the authors should avoid causal interpretations.

**Reply: We agree our finding is correlational in nature. The language we used is hopefully not too strong (e.g., the finding can “help lab leaders”) and we also now incorporate additional support from previous work that found relatively similar effects.**

**References**

 1. Powell K: The future of the postdoc. *Nature*. 2015; **520** (7546): 144-7 PubMed Abstract | Publisher Full Text 

 2. Larson RC, Ghaffarzadegan N, Xue Y: Too Many PhD Graduates or Too Few Academic Job Openings: The Basic Reproductive Number R0 in Academia. *Syst Res Behav Sci*. **31** (6): 745-750 PubMed Abstract | Publisher Full Text 

 3. Ghaffarzadegan N, Hawley J, Larson R, Xue Y: A Note on PhD Population Growth in Biomedical Sciences. *Syst Res Behav Sci*. **23** (3): 402-405 PubMed Abstract | Publisher Full Text
